# Public spending on immunisation in Poland

**DOI:** 10.3389/fpubh.2025.1682169

**Published:** 2025-12-05

**Authors:** Michał Seweryn, Joanna Augustyńska, Justyna Kopel, Rafał Jaworski, Alicja Wójcik, Iwona Paradowska-Stankiewicz, Marcin Czech

**Affiliations:** 1 Faculty of Medicine, Andrzej Frycz Modrzewski Krakow University, Krakow, Poland; 2 EconMed Europe, Krakow, Poland; 3 MSD, Warsaw, Poland; 4 Department of Epidemiology of Infectious Diseases and Surveillance, National Institute of Public Health NIH - National Research Institute, Warsaw, Poland; 5 Pharmacoeconomic Department, Institute of Mother and Child, Warsaw, Poland

**Keywords:** vaccinations, vaccines, immunisation, costs, public payer expenditure, immunisation cost

## Abstract

**Objective:**

Vaccination is a proven method for preventing infectious diseases and their complications at individual and community levels. This study aimed to estimate actual public spending on immunisation and compare it with expenditures in other categories of direct medical costs and indirect costs related to absenteeism.

**Methods:**

The analysis was based on previously published modelling framework *Immunisation Planning Tool (IPT 2.0)*, populated with vaccine acquisition and administration cost data, vaccination coverage, demographic characteristics, and the scope of the immunisation programme, which defines the recommended schedules for specific patient populations in Poland. Spending on immunisation was compared with total health expenditure, budgets for drugs and prevention, and more specific expenditure categories (e.g., common diseases and drug groups classified by ATC system). In addition, it was compared with indirect costs related to absenteeism due to vaccine-preventable diseases.

**Results:**

The annual healthcare budget in 2024 in Poland was €44,752 million, of which €6,087 million (13.6%) was allocated to drug and €769 million (1.7%) to prevention. The immunisation programme cost €206 million (0.5%), with vaccine acquisition accounting for 91% of total immunisation costs. The annual per capita cost of vaccines was €4.99, which was notably lower than per capita reimbursement costs for drug classes used to treat diabetes (€12.12), cardiovascular diseases (€12.67), nervous system diseases (€9.42), and respiratory diseases (€7.68). Additionally, the total cost of absenteeism due to vaccine-preventable diseases (€371 million) significantly exceeded the total annual expenditure on the immunisation programme in Poland.

**Conclusion:**

With 1.7% of its healthcare budget allocated to prevention and 0.5% to vaccinations, Poland remains a country that invests relatively little in preventive measures. Given the high return on investment in immunisation—through reductions in both direct and indirect costs and in severe outcomes—increased and sustainable public funding for vaccination should be prioritised by healthcare policymakers. Sustainable public investment is crucial to addressing key immunisation challenges: low coverage, delayed introduction of cost-effective vaccines and service quality gaps. Rather than being driven by international expenditure comparisons, this investment should be justified by the evidence-based potential of immunisation to reduce the health and economic burden of vaccine-preventable diseases.

## 1 Introduction

Vaccination is one of the key preventive interventions and a cornerstone of public health policy. In 1974, the World Health Organization launched the Expanded Programme on Immunisation. Since then, vaccination has prevented 154 million deaths, resulting in a gain of 10.2 billion years of full health (1). Most vaccines offer protection in two ways: they directly reduce the risk to the vaccinated individual and, in most cases, they help decrease community transmission and overall exposure to infectious diseases (1). As a public health measure, vaccination provides additional benefits, including increased productivity, lower healthcare resource utilisation, and reduced antibiotic resistance (2).

In Poland, the first vaccination programme was introduced in the 1960s, initially covering variola, tuberculosis, diphtheria, tetanus, and pertussis (3). Substantial evidence supports the success of the national vaccination programme and its positive impact on public health in Poland. Research has shown that the Paediatric Immunisation Programme (part of the National Immunisation Programme aimed at children) significantly reduces disease burden and risk of premature death. It was estimated to have prevented approximately 452,300 disease cases and 1,600 premature deaths in the Polish 2019 birth cohort (4). Furthermore, the programme enables substantial cost savings from both healthcare and societal perspectives by reducing disease incidence and associated expenses. Every €1 invested in the Paediatric Immunisation Programme generates a return of more than €2 from the healthcare payers’ perspective and more than €7 from the societal perspective (4).

Recently, new vaccines have been included in public funding in Poland, offering promise for further improvements in public health outcomes by reducing the consequences of infections caused by pneumococci, rotaviruses, influenza viruses, herpes zoster viruses, and human papillomaviruses (HPVs) (5, 6).

At the same time, it should be acknowledged that public discourse around vaccination in Poland is influenced not only by medical and economic considerations, but also by broader social and political dynamics. The activities of a strong anti-vaccination movement, closely linked with right-wing populist groups, has contributed to increasing parental resistance and a growing share of unvaccinated children even prior to the COVID-19 pandemic (7). This atmosphere has been further amplified by online misinformation and anti-vaccine narratives spread through digital platforms such as YouTube (8).

Vaccination appears to be the health technology with the highest return on investment. Infectious diseases, including those preventable by vaccination, impose a substantial economic burden to society (9); therefore, estimating the real costs of implementing immunisation programmes in Poland and assessing the adequacy of allocated resources would offer valuable insights for future healthcare budget planning. This analysis compared immunisation spending with other healthcare-related expenditures and the costs of absenteeism due to such infectious diseases.

## 2 Materials and methods

### 2.1 Study design

The cost modelling study was based on the previously published modelling framework developed by Bencina et al. (10); *Immunisation Planning Tool (IPT 2.0)*, which estimates the individual lifetime costs of vaccination with the vaccines recommended and funded in 23 European countries, including Poland. As part of our analysis, the model was populated using Polish-specific data to cover mandatory and recommended vaccinations funded in Poland as well as to include new vaccines that were not initially considered in the previous analysis. These include recommended vaccines fully financed by the Ministry of Health (MoH), such as the HPV vaccine, coronavirus 2019 (COVID-19) vaccine, and pertussis vaccine for pregnant women, which represents an update compared to 2022 analysis published by Bencina et al. (10). Mandatory vaccination refers to the legal obligation for individuals to receive vaccinations as part of the National Immunisation Programme. Non-compliance with this obligation may result in penalties, such as fines, imposed by relevant authorities. Additionally, the model included vaccines that are partially or fully reimbursed through the pharmacy chain based on prescription, including the HPV vaccine, pneumococcal conjugate vaccine (PCV) for adults, and herpes zoster vaccines for adults, as well as respiratory syncytial virus (RSV) immunisation provided through a reimbursed drug programme. Vaccines included in the current analysis represents immunisation schedule in 2024.

The analysis was conducted from the public payer’s perspective, as the public payer covers the majority of vaccine costs in Poland, with citizen co-payment representing a relatively small share. The costs of fully financed mandatory vaccinations and recommended vaccinations that are fully or partially financed by the list of reimbursed medicines, are covered by the National Health Fund (NHF). Funding for the acquisition of recommended vaccines, which are fully financed by decision of the MoH, comes primarily from the MoH budget and partly from the NHF. The cost assessment included expenses related to vaccine acquisition and administration costs for specific immunisation schedules.

In addition, the healthcare and drug budgets for 2024 were estimated. The value and share of expenditures on prevention were also determined to assess the percentage of spending on immunisation. Costs were collected and estimated in Polish zloty (PLN) and converted to euros (€) using the 2024 yearly average exchange rate of €1 = PLN 4.3064 (11).

### 2.2 Variables and data sources

#### 2.2.1 Healthcare expenditure

Estimates of current public payer expenditures (2024) for total healthcare and drug budgets were based on the financial plan of the NHF (12). The cost categories included primary healthcare, outpatient and inpatient care, psychiatric care, medical rehabilitation, palliative and hospice care, emergency and sanitary transport, medical rescue, etc., as well as drugs and vaccines. The total healthcare budget also included costs directly incurred by the MoH for medical services, drugs, and vaccines. Thus, the total healthcare budget encompassed also drug-related expenses such as reimbursed drugs sold in pharmacies, drugs used in drug programmes and chemotherapy, and those provided through emergency access to drug technology (13).

In Poland the Ministry of Health (MoH) is responsible for establishing national healthcare policy, strategic planning, funding certain services, and coordinating funding at the central level, while the NHF manages the financing and reimbursement of healthcare services. Local governments, on the other hand, are responsible for the implementation and delivery of healthcare services at the regional and municipal levels, including funding for additional services not covered by the central government.

The actual total spending on prevention in Poland is difficult to estimate, as it is financed through both the central budget and local government budgets. These costs are not fully reflected in the NHF budget, which includes only spending on prevention programmes (such as cancer screening tests) and vaccination programmes. Prevention-related expenditure is actually distributed across various funding sources, making it difficult to capture their full scale. Expenditures on preventive healthcare by local governments are not fully disclosed to the public, making it impossible to precisely determine their total amount. Due to differences in financing and categorising costs, it was not feasible to timely gather the relevant data from all these institutions.

Prevention expenditures are also reported by the Organisation for Economic Co-operation and Development (OECD) (14), which includes components such as immunisation, early disease detection, and epidemiological surveillance. However, OECD data is not fully reliable for our analysis, as it often omits recent funding mechanisms for vaccines available in pharmacies (e.g., HPV, pneumococcal) and does not account for specific public health measures like RSV vaccination for high-risk infants. These gaps in data make the OECD figures incomplete for estimating the total prevention expenditures in Poland.

Given the uncertainties in data for Poland reported to the OECD and difficulties in estimating costs of prevention from publicly available Polish sources, this analysis employed an alternative approach to estimate the costs of prevention. The costs of the immunisation component were based on estimates from the updated modelling framework by Bencina et al. (10), while the costs of the other components (information, education and counselling programmes, early disease detection programmes, healthy condition monitoring programmes, epidemiological surveillance and risk and disease control programmes, preparing for disaster preparedness and emergency response programmes) were projected using linear regression of expenditures for the years 2018–2022 (14). A linear regression model using the method of least squares was applied to estimate future expenditures based on historical data. In this model, time (year) was treated as the independent variable, while the annual public payer costs were treated as the dependent variable. The regression equation is as follows:


y=β×x+ε

Where y represents the public payer costs, x is the time variable (year), β is the coefficient of the time variable, and ϵ is the error term. Calculations were initially performed in Polish złoty and subsequently converted to euros using the 2024 exchange rate. The model assumes that expenditures increase linearly over time, reflecting the historical upward trend observed during the reference period. This approach allows for the prediction of future expenditures, assuming that the trend observed in the past will continue in a similar manner.

#### 2.2.2 Vaccines acquisition costs

**Figure 1:**
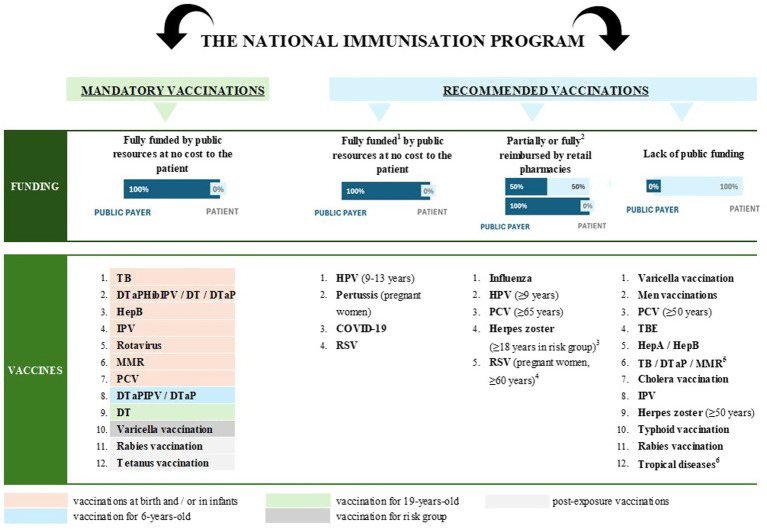
The funding system for vaccinations in Poland. DT, diphtheria and tetanus; DTaP, diphtheria, tetanus, and acellular pertussis; HepA, hepatitis A; HepB, hepatitis B; Hib, haemophilus influenzae type B; HPV, human papillomavirus; IPV, inactivated polio vaccine; MMR, measles, mumps, and rubella; PCV, pneumococcal conjugate vaccine; RSV, respiratory syncytial virus; BCG, tuberculosis; TBE, tick-borne encephalitis.^1^Minister of Health’s decision on the funding of selected recommended vaccinations for specific patient groups during a designated period of time; ^2^Fully reimbursed for patients under 18 years of age (HPV, influenza), over 65 years of age (influenza, pneumococcal vaccine), and pregnant women (influenza); ^3^The herpes zoster vaccine has been reimbursed for patients aged 18 and older in risk groups since April 2025, whereas until March 2025, it was reimbursed for patients aged 65 and older in risk groups, and this population was included in the estimation for 2024; ^4^Vaccination not included in estimates, reimbursed from April 2025; ^5^Recommended vaccinations for previously unvaccinated patients and individuals in risk groups; ^6^Japanese encephalitis, yellow fever, dengue fever, mpox disease.

In line with the chosen perspective, only the public payer costs of implementing vaccination programmes were included. The National Immunisation Programme in Poland (15) is divided into mandatory vaccinations (fully financed by public funds) and recommended vaccinations (Figure 1), some of which are fully or partially funded by the public payer. The recommended HPV vaccine for individuals aged 9–13, the COVID-19 vaccine, and the pertussis vaccine for pregnant women are fully funded following a decision by the MoH. In addition, four recommended vaccines, including HPV (for individuals aged 9 years or older, as per the Summary of Product Characteristics) (16), influenza, pneumococcal vaccine for adults, and herpes zoster, are reimbursed for patients through the pharmacy channel based on prescription, with a maximum patient co-payment of 50%. Pharmacy reimbursement is based on prescriptions issued by physicians, although recently pharmacists have also been entitled to issue prescriptions for vaccines.

The RSV vaccine is funded separately, in accordance with the criteria outlined in the fully reimbursed drug programme. The “RSV Infection Prevention” programme is intended for patients in the following risk groups: neonatal patients; patients diagnosed with cystic fibrosis up to 1 year of age; cardiac patients up to 2 years of age; and patients with spinal muscular atrophy up to 2 years of age. The average number of administrations and the average cost of the vaccine (depending on patient weight) and its administration were estimated using 2023 data (17). Vaccination coverage in the general population of children under 2 years of age was also estimated based on these data. Post-exposure vaccinations were not included in further calculations because they are administered in response to exposure to a pathogen.

Depending on the reimbursement method, vaccine acquisition costs in 2024 were derived from publicly available sources including MoH tenders (18) for the procurement of mandatory and recommended vaccines fully financed from public funds, as well as from the NHF’s reimbursement expenditures for vaccines available in pharmacies according to the list of reimbursed medicines (Table 1). The cost of certain vaccines (e.g., diphtheria and tetanus, COVID-19) depends on the population in which the vaccine is administered, including factors such as age group and contraindications to vaccination. Unit costs, including value-added tax, were presented as the cost of a single vaccine dose based on 2024 data.

**Table 1:** Cost per dose of vaccine acquisition and administration in Poland in 2024 (17–19, 42, 43).

Vaccine	Number of doses	Vaccine acquisition cost/administration cost
BCG	1	€20.39/–
DTaPHibIPV	4	€27.27/–
DTaP	4	€11.86/–
DT^1^	4	€147.21/–
IPV	4	€9.53/–
Hib	4	€10.41/–
HebB	4	€3.53/–
Rotavirus	2	€11.28/–
MMR	2	€10.22/–
PCV	3	€13.02/–
DTaPIPV^2^	1	€14.57/–
RSV	2.8	€671.69/€45.17
DT^3^	1	€8.03/–
Varicella	2	€26.82/ -
Influenza (pharmacy)	1/year	€10.75/€0.72
HPV (pharmacy)	2.8	€59.79/–
HPV	2	€77.18/€13.50^4^
PCV (pharmacy)	1	€65.05/€7.75
Zoster (pharmacy)	2	€87.20/–
Pertussis (pregnancy)	1	€9.98/–
COVID-19 (<12 years)	1	€60.05/€7.75
COVID-19 (≥12 years)	1	€27.81/ €7.75
COVID-19 (pregnancy)	1	€30.09/€7.75

DT, diphtheria and tetanus; DTaP, diphtheria, tetanus, and acellular pertussis; HepB, hepatitis B; Hib, Haemophilus influenzae type B; HPV, human papillomavirus; IPV, inactivated polio vaccine; MMR, measles, mumps, and rubella; PCV, pneumococcal conjugate vaccine; RSV, Respiratory syncytial virus; BCG, tuberculosis.

^1^Vaccination for infants with contraindications to pertussis vaccination; ^2^Vaccination for 6-year-olds; ^3^Vaccination for 19-year-olds; ^4^The cost of administering the vaccine is higher when delivered in a school facility than in a primary care setting.

#### 2.2.3 Vaccine administration costs

In Poland, vaccines are typically administered as part of primary healthcare visits, funded through a capitation payment that is independent of the number of visits per year. The annual capitation payment is allocated per patient registered with a given primary care physician and nurse (base capitation payment: €62.22), with higher rates assigned for children (€72.22–147.21), older population (€79.22–167.20) and patients with chronic conditions (€172.20), such as diabetes (19).

Recently, the administration of several vaccines has received dedicated additional funding: COVID-19; influenza for individuals aged ≥65 years; pneumococcal vaccines for individuals aged ≥65 years; and HPV vaccines for individuals aged 9–13 years.

The administration of the COVID-19 vaccine is additionally funded (€7.79), with the vaccination available at designated pharmacy points or health centres (20). Similarly, the administration of HPV vaccines (except those purchased at pharmacies) is also financed and can be carried out either at a health centre (€7.75) or at a school facility (€19.26). Due to the lack of available data on the distribution of HPV vaccines between health centres and school facilities, a conservative assumption of equal distribution was adopted. Consequently, the average cost of administering an HPV vaccine was estimated at €13.50 (Table 1). Based on available data, 9% of influenza vaccinations were administered at pharmacy points (21), so the average cost of administering this vaccination per patient was estimated at €0.72 (Table 1).

#### 2.2.4 Vaccination coverage

**Figure 2:**
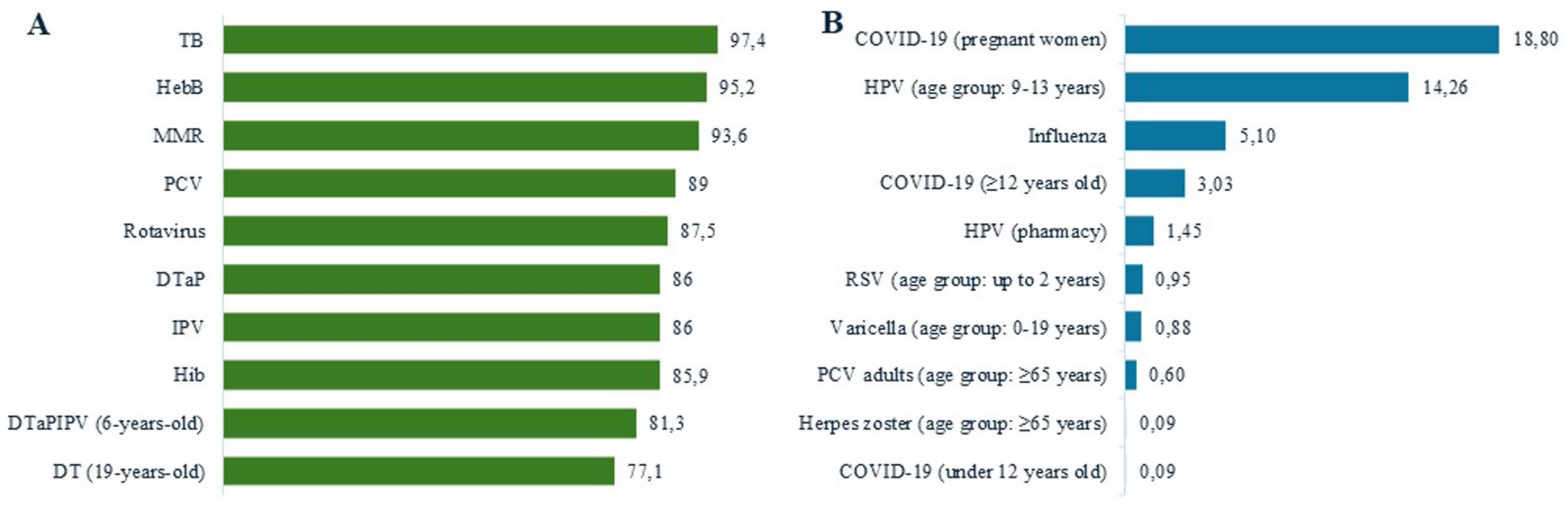
Vaccination coverage (%)—**(A)** mandatory vaccines^24^; **(B)** recommended or in risk-group vaccines^17,18,20,24^. DT, diphtheria and tetanus; DTaP, diphtheria, tetanus, and acellular pertussis; HepB, hepatitis B; Hib, Haemophilus influenzae type B; HPV, human papillomavirus; IPV, inactivated polio vaccine; MMR, measles, mumps, and rubella; PCV, pneumococcal conjugate vaccine; RSV, Respiratory syncytial virus; TB, tuberculosisHPV (pharmacy) – refers to patients aged 14 to 25 years, assuming that younger individuals are primarily vaccinated under the National Immunisation Programme; the upper age limit corresponds to the eligibility criteria used in clinical trials for the reimbursed product.

The number of doses was taken from the National Immunisation Programme (or the Summary of Product Characteristics if indicated as the source), while the coverage rates for mandatory vaccinations in the entire population were obtained from the annual *Vaccinations in Poland* report (22) (Table 1, Figure 2). For recommended vaccinations, it was assumed that vaccines purchased by the MoH would be fully utilised (e.g., for COVID-19), adopting a conservative assumption of maximum vaccination rates to ensure that vaccination costs were not underestimated. Due to varying vaccine prices by target population (Table 1), vaccination coverage is also presented by dividing patients into three groups: paediatric patients under 12 years of age, pregnant women, and other patients aged 12 and above. The model design required vaccination coverage in risk groups (varicella, adult pneumococcal, herpes zoster, RSV) to be estimated for the entire age group. In the absence of data, pertussis vaccination coverage for pregnant women was assumed to be equivalent to coverage in infants.

#### 2.2.5 Demographic data

Population characteristics, including the total population size (23), sex and age breakdown, life expectancy (24), number of live births, and population projections, were obtained from the Central Statistical Office of Poland. The annual number of pregnancies was obtained from NHF data (25). The most recent data were used (as of the end of 2023 and 2024, where available) (Table 2).

**Table 2:** Parameters used in the analysis.

Parameter	Value
Demographic data of Poland, 2023 (25–27)	
Number of inhabitants^1^	37,532,044
Number of pregnant women	265,941
Number of live births	297,443
Life expectancy in men	74.7 years
Life expectancy in women	82.0 years
Value of one working day in 2023—estimation	
Gross domestic product in Poland	€789,896 million
Number of individuals employed	17,323,000
Correction factor	0.65
GDP per employed individual, considering the CF	€29,639
Number of working days including holidays	227
Value of one working day	€130.57
Indirect costs (human capital approach), 2023	
Value of one working day	€130.57
Number of sickness absenteeism days	4,413,264
Value of productivity loss due to vaccine-preventable disease^2^	€576,228,128
Number of sickness absenteeism days excluding COVID-19	1,986,811
Value of productivity loss due to vaccine-preventable disease excluding COVID-19	€259,412,622

^1^Data from 2024; ^2^ICD-10 codes: A08 Viral and other specified intestinal infections; A09 Diarrhea and gastroenteritis of presumed infectious origin; A36 Diphtheria; A37 Whooping cough; B01 Varicella (chickenpox); B02 Zoster (herpes zoster); B05 Measles; B06 Rubella (German measles); B16 Acute hepatitis B; B26 Mumps; J9 Influenza due to identified zoonotic or pandemic influenza virus; J10 Influenza due to identified seasonal influenza virus; J11 Influenza, virus not identified; U07 COVID-19.

#### 2.2.6 Other costs

The costs of vaccine acquisition were compared with the costs of different groups of drug reimbursement classified according to the Anatomical Therapeutic Chemical (ATC) system (26): Alimentary tract and metabolism (including anti-diabetic drugs), Cardiovascular system, Nervous system, and Respiratory system (R) per capita. Drug reimbursement costs for 2023 were based on data from the NHF (27).

The costs of immunisation were also compared with the overall costs of treatment of common diseases such as diabetes (28), asthma (29), and ischaemic stroke (30) incurred by NHF in 2023. These conditions were chosen for two main reasons. Firstly, they represent significant public health challenges in the Polish population. Secondly, we have access to the most reliable and up-to-date data on the costs of these diseases in the Polish context, which aligns with the principle of using the best available evidence. The cost of diabetes care included the reimbursement of healthcare services (outpatient care, inpatient care, etc.), drugs, and medical devices used to treat diabetes (blood glucose test strips, pen needles, infusion pump–related products, and continuous glucose monitoring systems). Expenses related to asthma included outpatient and inpatient care, rehabilitation, and reimbursed asthma medications. The costs of stroke treatment included the costs of hospital care (such as mechanical thrombectomy and thrombolytic therapy) as well as the costs of neurological rehabilitation.

#### 2.2.7 Indirect costs

To broaden the scope of the comparison, indirect costs were considered to compare direct public spending on immunisation with societal costs related to absenteeism in 2023. This was based on sick leave attributed to diseases preventable by the vaccines included in our analysis. Sick leave associated with the following vaccine-preventable diseases, identified by ICD-10 codes, were considered: A08 Viral and other specified intestinal infections (number of sick leave certificates per year: 61,690); A09 Diarrhea and gastroenteritis of presumed infectious origin (127,068); A36 Diphtheria (11); A37 Whooping cough (165); B01 Varicella (chickenpox) (14,693); B02 Zoster (herpes zoster) (31,213); B05 Measles (30); B06 Rubella (German measles) (146); B16 Acute hepatitis B (65); B26 Mumps (262); J9 Influenza due to identified zoonotic or pandemic influenza virus (232); J10 Influenza due to identified seasonal influenza virus (37,832); J11 Influenza, virus not identified (160,887); and U07 COVID-19 (387,528). The inclusion of code J09 was dictated by the reporting of sickness absence days despite the lack of identified zoonotic or pandemic influenza cases in Poland, which suggests that the reported sickness absence days refer to other cases of influenza (e.g., seasonal influenza) (31). Indirect costs were calculated using data from Statistics Poland (32, 33), including gross domestic product (GDP), the number of employees, and information from the Social Insurance Institution database (34) on the number of sick leave days, applying the human capital approach.

The calculation of productivity lost was done according to Polish recommendations (35). In the human capital method, the estimation of indirect costs is based on calculating the value of lost production resulting from absenteeism or presenteeism (being at work but with reduced productivity) caused by illness. Our analysis includes the costs incurred due to patient absenteeism from work as a result of infectious diseases. The costs of lost productivity are estimated by determining the value of one working day. This value is calculated based on GDP current prices (31) per employed person (33), which is then multiplied by a correction factor of 0.65 (35). This factor allows for a more realistic estimate of productivity losses resulting from job loss, eliminating the influence of capital and other factors not directly related to employment. Then, based on the number of working days in a given year, excluding days allocated for annual leave (on average 23 days), the cost of one working day in Poland for 2023 was estimated to be €130.57 (Table 2). The indirect costs were calculated by multiplying the value of one working day by the number of sickness absenteeism days.

#### 2.2.8 Presentation of results

The results were presented as the costs of the immunisation programme (including the costs of acquiring and administering vaccines) and, separately, as the costs of vaccines (vaccine acquisition only). Outputs include categories such as total annual cost, cost per capita, cost per live birth, and lifetime immunisation cost per person (with distinctions between males and females), calculated separately for the risk groups. These risk groups included: individuals up to 19 years of age with immunodeficiency, those in contact with such patients, or those residing in care facilities or social care institutions (varicella vaccination); adults over 65 years of age at moderate to high risk of pneumococcal disease (pneumococcal vaccination); individuals aged 65 years and older at increased risk of developing herpes zoster (herpes zoster vaccination); and neonatal patients, infants with cystic fibrosis, infant cardiac patients, and infants with spinal muscular atrophy (RSV vaccination). Per capita costs were calculated assuming a population of 37.5 million in Poland in 2024 (Table 2).

## 3 Results

### 3.1 Public healthcare expenditure

**Figure 3:**
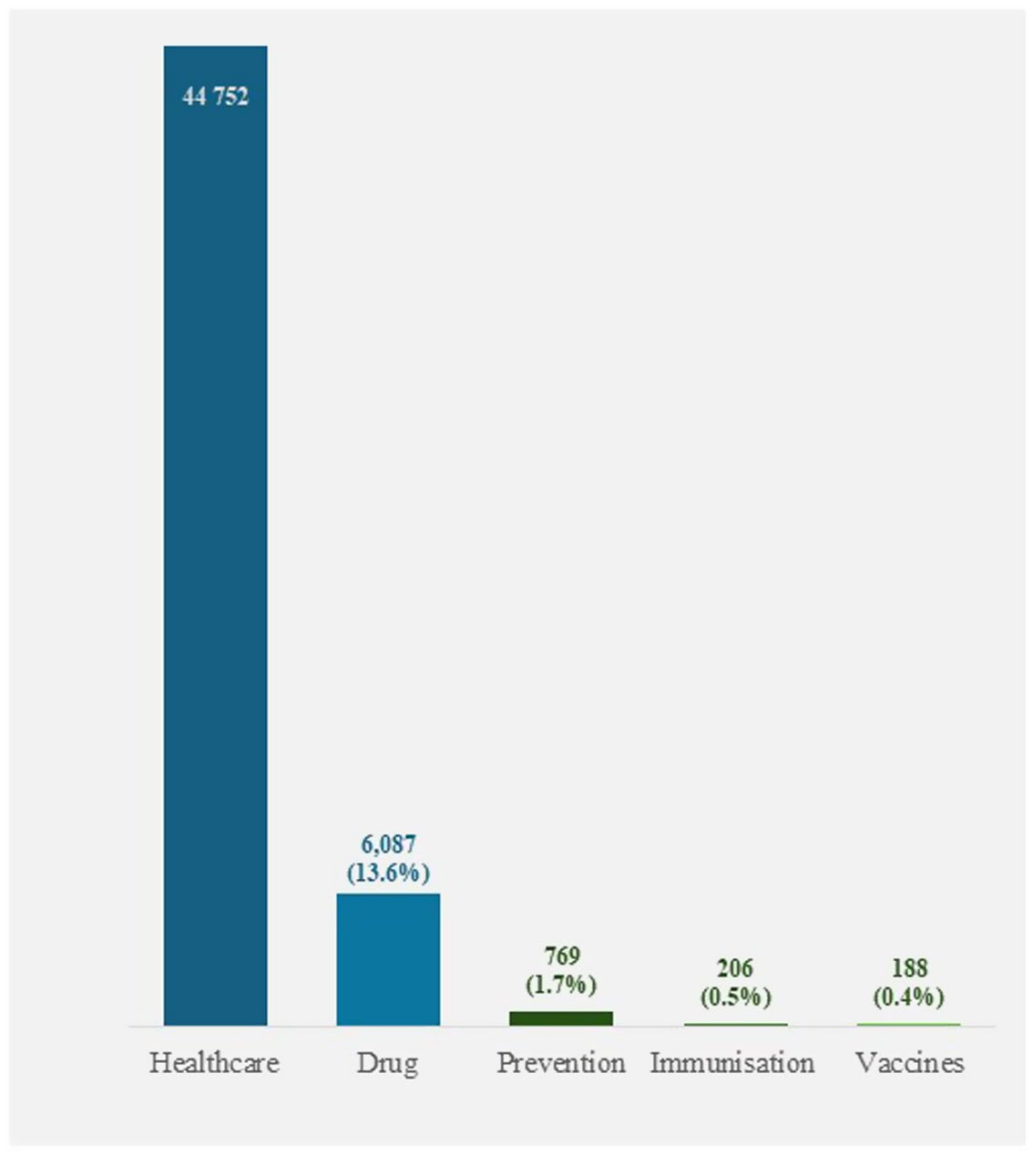
Healthcare spendings in Poland in 2024 (in million €). Total healthcare expenditure in Poland in 2024, including drug and prevention spending (in million €): total healthcare, drug, and prevention costs (including immunisation and vaccines), expressed as a percentage of total public expenditure on healthcare.

The total annual public healthcare budget in Poland for 2024 was €44,752 million, with €6,087 million allocated to the drug budget, accounting for 13.6% of the total expenditure (Table 3, Figure 3). Including the costs of immunisation (Table 4), the total costs of prevention reached €769 million, representing 1.7% of the total government expenditures on public health.

**Table 3:** Healthcare spendings in 2024.

Parameter	Annual cost (in million €)
Total public healthcare budget	44,752 (100%)
Public drug budget (% in total budget)	6,087 (13.6%)
Public prevention budget (% in total budget)	769 (1.7%)

The total healthcare and drug budgets were based on the final financial plan of the NHF (16); additionally, the total budget included costs incurred by the Ministry of Health (16). Public prevention spending was calculated by adding the projected prevention expenditure based on OECD data, excluding immunisation components (11), to the estimated immunisation programme costs for 2024 (Table 4).

**Table 4:** Annual immunisation costs in 2024—results from *Immunisation Planning Tool (IPT 2.0).*

Parameter	Annual cost (in €)
Total cost of immunisation programme	205,593,582
Total cost of vaccines	187,517,658
Cost of immunisation programme per capita	5.48
Cost of vaccines per capita	4.99
Cost of immunisation per live birth	691
Cost of vaccines per live birth	630

### 3.2 Spending on immunisation and vaccines

The cost of immunisation programme in 2024 (Table 4) was estimated at €206 million, the vast majority of which was the cost of acquiring vaccines, i.e., €188 million (91%). The immunisation programme budget represented less than 0.5% (Figure 3) of the total government expenditures on public health and 3.4% of the drug budget in Poland in 2024.

The cost of lifetime immunisation per capita and total lifetime cost of vaccines was slightly lower for men (€1,356 and €1,313, respectively) than for women (€1,404 and €1,353, respectively) (Table 5). This was mainly due to differences in life expectancy and, to some extent, due to additional spending on vaccines recommended for pregnant women (pertussis).

**Table 5:** Costs over the entire lifetime of patients—results from *Immunisation Planning Tool (IPT 2.0)*.

Parameter	Lifetime costs (in €)	
	Women	Men
Cost of lifetime immunisation per person	1,404	1,356
Cost of vaccines throughout life	1,353	1,313
Cost of lifetime immunisation per person in the risk group	3,694	3,646
Cost of vaccines throughout life in the risk group	3,514	3,473

The maximum lifetime immunisation costs for risk group patients amounted to €3,694 in women and €3,646 in men, respectively (Table 5). Vaccine acquisition costs were €3,514 and €3,473 for women and men, respectively. These maximum immunisation or vaccine costs were almost three times higher than those for the general population.

The estimated annual per capita cost of immunisation was €5.48 (Table 6), which was lower than the annual per capita costs of treating diabetes (3.8-fold higher), ischaemic stroke (2.1-fold higher), or asthma (1.4-fold higher) (Table 6). Additionally, the per capita reimbursement costs for groups of medicines used to treat diabetes (€12.12), cardiovascular diseases (€12.67), nervous system (€9.42), or respiratory diseases (€7.68) remained higher than costs allocated to vaccine funding (€4.76) (Table 6).

**Table 6:** Costs of public funding of selected drug groups and common diseases per capita (in €).

Parameter	Total annual cost	Cost per capita
Immunisation programme	205,593,582	5.48
Diabetes	774,366,060	20.63
Ischaemic stroke	426,201,636	11.36
Asthma	296,284,600	7.89
Vaccines/drugs (ATC system)		
Vaccines	187,517,658	4.99
A: Alimentary tract and metabolism	454,830,164	12.12
C: Cardiovascular system	475,388,031	12.67
N: Nervous System	353,703,073	9.42
R: Respiratory system	288,426,971	7.68

ATC system, Anatomical Therapeutic Chemical system (23).

Data sources: NHF databases—Immunisation and vaccine—Drug costs (24); Diabetes costs (25); Ischaemic stroke costs (27); Asthma costs (26).

### 3.3 Societal costs of infectious diseases

The number of days of absence due to vaccine-preventable diseases in 2023 was more than 4.4 million, with 2.4 million days for COVID-19 and more than 1 million days for influenza. Assuming the estimated average value of one working day in 2023 at €130.57, the value of productivity loss due to absenteeism from vaccine-preventable diseases was €576.2 million. In addition, the costs of productivity loss excluding sick leave caused by COVID-19 amounted to €259.4 million. These indirect costs of absenteeism significantly surpass the total annual expenditure on the immunisation programme in Poland. Considering sick leave due to COVID-19, the costs resulting from absenteeism were higher by €371 million. (Table 2)

## 4 Discussion

Our analysis confirms that the cost of the immunisation programme in Poland remains relatively low compared to the overall public healthcare budget. In 2024, the immunisation programme accounted for less than 0.5% of total government expenditures on public health, while the cost of vaccines accounted for 3.1% of the drug budget. The annual per capita cost of immunisation (€5.22) is substantially lower than per capita spending on treatment for some chronic diseases. The number of patients treated in 2023 for conditions such as diabetes, ischaemic stroke and asthma was 3.3 million (28), 74 thousand (30) and 2.1 million (29), respectively. In total, this resulted in 5.4 million patients (14% of total population) treated and over €1,500 million spent. However, the annual cost of immunisation was €206 million, which is only 14% of the cost of treating the above diseases. Importantly, the national vaccination programme reduces not only the risk to vaccinated individuals but also reduces community transmission and exposure to infectious diseases (1).

Lifetime vaccination costs were slightly higher for women than men, reflecting differences in life expectancy and maternal immunisation recommendations. The highest lifetime immunisation costs were observed in risk groups (up to €3,609).

Studies have shown that sickness absence due to infectious diseases represents a significant loss to the economy, estimated in Poland at €5.3 billion over a 6-year horizon (9). Notably, the indirect costs of adult sickness absence due to vaccine-preventable diseases (€576.2 million) far exceed the current spending on immunisation. This underscores the economic justification for further investment in vaccination programmes, not only to improve public health but also to reduce productivity losses. It is important to note that vaccines for diseases like COVID-19 and influenza are not 100% effective. However, they very reliably protect against the most severe outcomes of respiratory infections, including severe disease, hospitalization, and death. Although vaccinated individuals may still contract the illness or face variants not perfectly matched by the vaccine, the course of disease is typically significantly milder. Therefore, the benefits of vaccination, in terms of reducing the severity of illness and limiting the spread of disease, make it a crucial public health intervention. Despite the availability of publicly funded vaccines in Poland, coverage rates for some vaccinations remains suboptimal. Ensuring high uptake, particularly among vulnerable populations, and individuals with chronic conditions, requires tailored strategies following the best practices from other EU member states. Using an example from the United Kingdom; in 2022, the National Institute for Health and Care Excellence (NICE) issued recommendations to improve access to vaccination for the general population (36). Evidence showed that invitations or reminders to vaccinate were more effective than usual care in increasing vaccine uptake in all age groups. Different methods of contacting eligible individuals for vaccination are recommended, taking into account their preferences. For pregnant women, personal invitations to vaccination or offering vaccination at follow-up visits may be appropriate. However, for older population and individuals suffering from chronic diseases with limited access to outpatient clinics, or for residents of care institutions and similar facilities, vaccination should be made available through home visits. These measures also include invitations from trusted healthcare professionals, information on available vaccination sites, the option to book appointments online, efforts to raise patient awareness of the impact of vaccination on disease incidence (e.g., HPV) (36).

It is also recommended by NICE to increase vaccination uptake among older adults, extending HPV vaccination to boys, improving uptake of pertussis vaccination among pregnant women, introducing provider incentives, and involving schools and general practitioners in vaccination promotion (36).

In Poland, these proposals are implemented to a certain degree through the full or partial reimbursement of vaccines (e.g., influenza, PCV) in pharmacies for patients aged 65 years and older based on prescription. HPV vaccination is publicly funded for both boys and girls aged 9–13 years, with the vaccine being administered by general practitioner onsite or at school as an option. Healthcare providers receive additional funding for administering vaccines (COVID-19, HPV), whereas previously these vaccinations were covered by a fixed annual fee paid to primary care physicians. The pertussis vaccine has recently been reimbursed for pregnant women. From April 2025, RSV vaccines are reimbursed as prescription vaccine in pregnant women and in people aged 60 years and older. This marks another step in expanding the range of available free vaccinations for particularly vulnerable patient groups.

It’s important to notice that one of the probable reasons why the number of COVID-19-related deaths per million inhabitants in Poland was substantially higher than the EU average was the relatively low vaccination coverage (37). Despite the evidence of vaccines’ effectiveness, the pandemic also intensified social polarization regarding immunisation. As a result, a few years after its peak, around 20% of the population remain firmly opposed to vaccination, while up to 30% display varying levels of hesitancy (38).

It is important to continue investment in the immunisation and secure sustainable budget for vaccine funding. The broad promotional and educational campaigns to increase awareness on the benefits from vaccination and initiatives increasing vaccine coverage are also of great importance. In Poland, the possibility of school-based vaccination is likely to enhance access to publicly funded HPV vaccination and contribute to increased vaccination coverage. However, it is too early to assess this impact, as this option was only recently introduced (September 2024). The NICE concluded that vaccinating school-aged children and adolescents within the school setting is the most efficient and convenient way to vaccinate this population (36).

Expenditure on healthcare funded from the state budget is steadily rising. According to Eurostat, Poland allocated 5.7% of GDP to healthcare in 2023, and this would increase to 7% of GDP if private sector spending was included (39). For comparison, in 2018, health spending amounted to 4.5% of GDP in the public sector and 6.3% when both public and private sectors were included (39). According to OECD data, by 2022, public sector expenditure accounted for approximately 72–74% of total healthcare spending (14). Initial findings suggest that public sector participation had increased to over 80% by 2023 (14). In 2022, the budget for prevention in Poland totalled nearly €800 million, with just over 60% derived from public funds, amounting to €495 million. Total expenditure on prevention represented 1.9% of the overall healthcare budget, whereas within the public sector, it amounted to 1.6% in 2022 (14).

Despite a steady rise in per capita healthcare spending (expressed in euros per capita, adjusted for purchasing power parity [PPP]), Poland continues to invest slightly less than the average for other Central and Eastern European (CEE) countries: €1,908 vs. €2,098 in total healthcare spending, and €1,407 vs. €1,566 in public healthcare expenditure (14).

Notably, Poland ranks lowest in the region when it comes to spending on preventive care, allocating only €36 per capita from all sources and €23 from the public sector. This contrasts sharply with the CEE averages of €81 and €70, respectively. In terms of public budget expenditure on prevention, countries such as the Czech Republic (€129), Slovenia (€112) and Lithuania (€109) lead, investing five to six times more than Poland. Additionally, Estonia and Lithuania dedicate the highest share of their public healthcare budgets to prevention—6 and 7%, respectively—while Poland allocates only 1.6% (39). Over a span of 5 years (until 2022), public healthcare expenditures in CEE countries rose by 27–57%. During the same period healthcare spending in Poland increased by 33%, while spending on prevention rose by only 3%. Overall, countries in the CEE region experienced an average increase of 90% in prevention expenditure (14).

The median cost of immunisation in a study by Bencina et al. (10) referring to 2022 data, was estimated at €21 per person (ranging from €8 in Bulgaria to €43 in Germany). In contrast, immunisation expenses in 2024 in Poland were €5.5 per capita, highlighting that these costs were significantly lower than in other countries. OECD data (14) from 2022 further indicate that, among CEE countries, Poland likely has the lowest level of spending on immunisation (data for Hungary and Slovakia are unavailable). Among the other analysed countries, Latvia recorded the lowest spending on immunisation at €11.8 per capita (adjusted for PPP), while the Czech Republic reported the highest, at €55.9 per capita.

Bencina et al. (10), reported higher immunisation costs in Poland than our estimates because it assumes an administration cost of approximately €8.70 (40 PLN) for each vaccine dose, whereas our analysis applies the administration cost only to a limited number of vaccines against influenza, HPV, PCV, RSV, and COVID-19. In Poland, the reimbursement system does not provide additional fees for administering vaccinations, except for a few cases noted above. Primary care centres receive a fixed annual fee regardless of patient attendance or vaccination. As a result, it is difficult to fully estimate the actual costs of vaccine administration, which may constitute a limitation of the analysis. As a result, Bencina et al. (10), estimated somewhat higher expenses for the public payer (€9 per capita) compared to our estimate (€5.48), which appears to more accurately reflect the actual (2024) expenses associated with publicly funded immunisation programmes in Poland.

Conversely, OECD data (14) indicated that the vaccination cost per capita in Poland in 2022 was only €1, which seems implausibly low. This suggests that OECD data may be incomplete, likely covering only select cost categories. Additionally, it is worth highlighting that, according to OECD statistics, the highest reported per capita immunisation cost in Poland over the past 5 years (2018–2022) was €2.6 in 2018 (with no data available for 2020).

This analysis has some limitations. Accurately estimating prevention-related healthcare costs in Poland is challenging, as funding comes from both the central and local government and is spread across various budgetary sources. Although the OECD provides relevant data (14), certain components—such as costs of immunisation programmes—appear to be significantly underestimated; for instance, OECD figures are approximately four times lower than those reported by the NHF. To address these discrepancies, a combined approach for the costs of prevention was applied. The cost of the immunisation was estimated using the updated tool for 2024, while the costs of the other components were projected with linear regression based on OECD expenditure data from 2018 to 2022. In this model, time (year) was treated as the independent variable, and the dependent variable was the reported public expenditure on healthcare related to prevention. The regression allowed us to project future costs by identifying trends in historical data. This method ensures that the most reliable data, particularly those well-documented by the NHF, are incorporated, while also aligning the analysis with OECD figures for broader consistency.

Another limitation is the uncertainty associated with estimating vaccination coverage rates for recommended vaccines, particularly for products with no published data or where the available information changes dynamically, as is the case with COVID-19 vaccinations. As a result, a conservative assumption of maximum vaccination rates was adopted to avoid underestimating the costs incurred for vaccinations. It can be anticipated that the actual vaccine costs will be slightly lower than the estimated values.

Indirect costs do not include the cost of lost productivity due to absenteeism of parents of sick children, as the reported data do not allow identification of the diagnosis of the sick child. However, it should be noted that some of these were due to vaccine-preventable diseases (e.g., influenza, chickenpox, rubella), and with more than 13 million days (40) of absenteeism related to caring for a sick child (or adult family member) in 2023, the indirect costs are likely to be much higher than estimated. In addition, parental care allowances are paid from the Social Insurance Institution budget, so reducing the incidence of these conditions will reduce spending in this area.

The estimates of indirect costs account for absenteeism; however, presenteeism is equally significant during infectious illnesses. This refers to employees attending work while sick, often resulting in reduced productivity due to illness-related symptoms. A systematic review (41) found that 60–80% of employees with influenza or influenza-like illness continue to work while symptomatic, leading to lower productivity as they are present but not functioning at full capacity. The publication highlights the substantial impact of presenteeism on work productivity.

## 5 Conclusion

Our analysis reveals that the financial burden of the immunisation programme in Poland is relatively modest in relation to the overall public healthcare budget. In 2024, the immunisation programme accounted for less than 0.5% of total health expenditure, while the cost of vaccines made up 3.1% of the pharmaceutical budget 1.7% of total government expenditures on public health. In the context of the proven effectiveness and cost-effectiveness of vaccines in preventing severe disease, complications, hospitalisations and deaths, such a low share indicates underinvestment in a key public health intervention.

Despite immunisation being an intervention aimed at the general public, our analysis demonstrates that the per capita costs for vaccination in Poland are substantially lower than the public expenditures for each of the diseases selected for comparison, including diabetes, ischaemic stroke and asthma. Additionally, we highlighted the significant indirect costs (absenteeism cost) associated with infectious diseases that could be prevented through vaccination. Therefore, the argument for strengthening and increasing funding for immunisation in Poland should be grounded primarily in evidence-based needs, such as low and stagnating coverage rates, delayed introduction of cost-effective vaccines and gaps in service quality.

Taken together with low vaccination coverage rates, particularly among adults and adolescents, as well as the failure to introduce vaccines that are both cost-effective and responsive to Poland’s evolving epidemiological profile, these findings highlight the urgent need to prioritize vaccination in public health policy in Poland. It is critical to recognize the role of vaccination not only in preventing infectious diseases but also in reducing the economic burden on the healthcare system. To address these challenges, increasing the vaccination budget is necessary. In this context, higher and more stable public funding should be viewed as an instrument to unlock the proven potential of immunisation to prevent severe disease, hospitalisations and deaths, and to improve the efficiency and resilience of the healthcare system. It is also crucial to underline that responsibility for financing vaccination should primarily constitutes the state objective, since only sustainable public investment can support introduction of the new vaccines, increase coverage rates, and bring Poland to the standards of other EU member states increasing the share of the primary prophylaxis in the total health care expenditures.

Given the high return on investment in immunisation—through reduced treatment costs and productivity losses—increasing funding for vaccination is an evidence-based strategy that should be prioritised by policymakers. At the same time, it is important to acknowledge that the effectiveness of vaccination policy in Poland depends both on the level of financial resources allocated and different societal and political factors. The COVID-19 pandemic has had a profound impact on vaccination attitudes, with a considerable proportion of the population demonstrating vaccine hesitancy or outright opposition. These societal and political factors, coupled with the spread of misinformation, represent additional barriers that may limit the impact of purely financial measures. Therefore, while our study focuses primarily on the economic dimension of vaccination funding, the interpretation of our findings should also be considered taking into account these societal determinants, which will be crucial for designing effective and sustainable vaccination strategies in the future.

## Data Availability

The original contributions presented in the study are included in the article/supplementary material, further inquiries can be directed to the corresponding author.
